# Poaceae Pollen from Southern Brazil: Distinguishing Grasslands (Campos) from Forests by Analyzing a Diverse Range of Poaceae Species

**DOI:** 10.3389/fpls.2016.01833

**Published:** 2016-12-06

**Authors:** Jefferson N. Radaeski, Soraia G. Bauermann, Antonio B. Pereira

**Affiliations:** ^1^Universidade Federal do PampaSão Gabriel, Brazil; ^2^Laboratório de Palinologia da Universidade Luterana do Brasil–ULBRA, Universidade Luterana do BrazilCanoas, Brazil

**Keywords:** pollen morphology, grasses, pampa, South America, Atlantic forest, bamboo pollen

## Abstract

This aim of this study was to distinguish grasslands from forests in southern Brazil by analyzing Poaceae pollen grains. Through light microscopy analysis, we measured the size of the pollen grain, pore, and annulus from 68 species of Rio Grande do Sul. Measurements were recorded of 10 forest species and 58 grassland species, representing all tribes of the Poaceae in Rio Grande do Sul. We measured the polar, equatorial, pore, and annulus diameter. Results of statistical tests showed that arboreous forest species have larger pollen grain sizes than grassland and herbaceous forest species, and in particular there are strongly significant differences between arboreous and grassland species. Discriminant analysis identified three distinct groups representing each vegetation type. Through the pollen measurements we established three pollen types: larger grains (>46 μm), from the Bambuseae pollen type, medium-sized grains (46–22 μm), from herbaceous pollen type, and small grains (<22 μm), from grassland pollen type. The results of our compiled Poaceae pollen dataset may be applied to the fossil pollen of Quaternary sediments.

## Introduction

**Figure 1:**
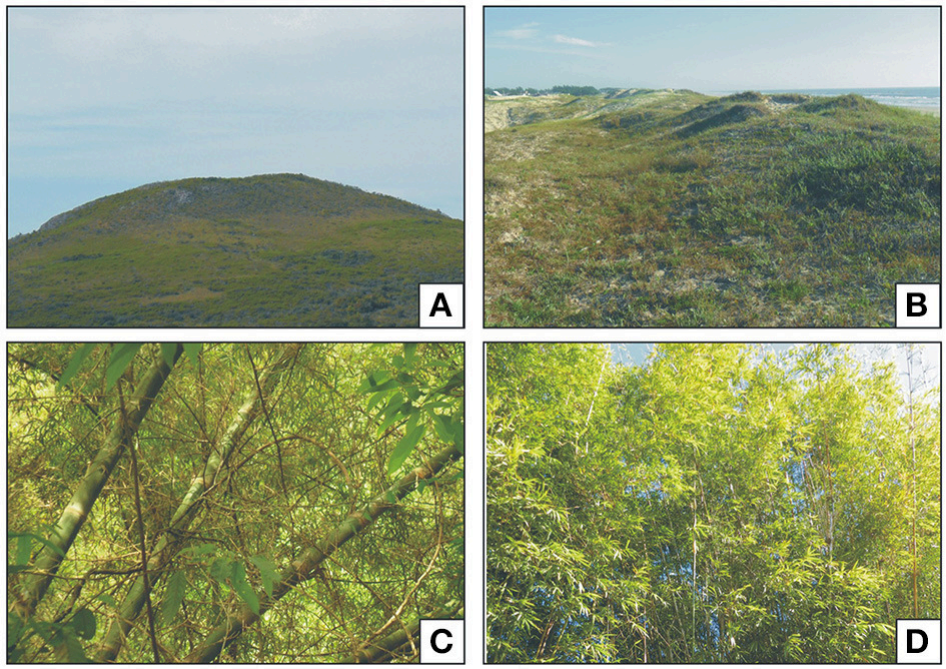
**(A)** Grassland vegetation of Rio Grande do Sul, “Cerro do Ouro,” São Gabriel city. **(B)** Grassland of the coastal plain of RS, “Balneário Quintão,” Palmares do Sul city. **(C,D)** Riparian forest with lignified bamboos, Gravataí city.

Pollen grains of the family Poaceae are widely found in Quaternary sediments of southern Brazil (e.g., Behling et al., 2004; Macedo et al., 2007; Bauermann et al., 2008). However, the stenopalynous nature of the pollen from this family makes it difficult to determine subfamilies and genera using pollen data (Erdtman, 1952; Salgado-Labouriau, 1973). Consequently, low taxonomic resolution hampers paleoecological inferences. Being mainly associated with grassland vegetation (Figure 1), Poaceae pollen is usually interpreted as indicative of open formations. However in Rio Grande do Sul (RS), where more than 80% of Poaceae species occupy grasslands, a significant percentage (20%) of representatives of this family inhabit forest vegetation (Boldrini and Longhi-Wagner, 2011).

Although the Poaceae family is well represented and studied in RS, few studies have examined the pollen representatives of this family. The few descriptions or illustrations of pollen representatives presented in the study by Tedesco et al. (1999), which analyzed the diameter of the pollen grains of *Hemarthria altissima* under different ploidy levels, noted that the average diameters were variable, depending on the ploidy. However, the analyzed pollen grains were not acetolyzed. Medeanic et al. (2008) illustrated images of pollen grains from nine species, while Wilberger et al. (2004) presented images of pollen grains corresponding to three separate species. Nakamura et al. (2010), addressing the development of anther and pollen grains in *Axonopus aureus, Chloris elata*, h *Eragrostis solida, Olyra humilis, Paspalum polyphyllum*, and *Sucrea monophylla*, found similar pollen morphology between taxa. This excludes those grains that have been observed to have patterns that are important for differentiation between species of the family. Radaeski et al. (2011) described the pollen morphology of *Paspalum notatum, Paspalum plicatulum*, and *Schizachyrium microstachyum*. Later, Bauermann et al. (2013) described the pollen grains of *Andropogon lateralis* and *Eragrostis bahiensis*. Radaeski et al. (2014a) described the pollen morphology of *Eragrostis neesii*, and Radaeski et al. (2014b) contributed to the pollen description of six taxa of Poaceae, which showed that, in general, the pollen grains are of average size, with a monoaperture and spherical forms, and noted the identified stenopalynous characteristic of the family.

In South America, studies have been conducted on Poaceae pollen grains from Venezuela, Chile, Brazil, and Argentina (Heusser, 1971; Markgraf and D'Antoni, 1978; Salgado-Labouriau and Rinaldi, 1990). In Chile, the overlap between dimensions of the pollen grains of some tribes made it difficult to differentiate between tribes or subfamilies (Heusser, 1971). Thus, further studies on the pollen grains from other taxa are needed. Likewise, the same is true of the pollen grains of Barro Colorado Island (Roubik and Moreno, 1991). However, analyses of many species that are based solely on one pollen grain cannot verify the variations in total pollen grain diameter of the species. Analyzing pollen grains from Argentina, Markgraf and D'Antoni (1978) observed—from other grassland species studied—the largest diameter of pollen grain of the bamboo *Chusquea culeou*. Thus, analysis of pollen grains from other forest species can provide patterns related to vegetation from this site.

The pollen grains of many species from other regions of the world (e.g., Europe, South America) have been analyzed by scanning electron microscopy (SEM). Some of these species also inhabit southern Brazil. Some studies (Table 1) have explored the surface of the Poaceae pollen grains through SEM, which has contributed to separation of taxonomic groups (Köhler and Lange, 1979; Linder and Ferguson, 1985; Chaturvedi et al., 1994, 1998; Chaturvedi and Datta, 2001; Skvarla et al., 2003; Datta and Chaturvedi, 2004; Liu et al., 2004, 2005; Perveen, 2006; Kashikar and Kalkar, 2010; Ahmad et al., 2011; Dórea, 2011; Perveen and Qaiser, 2012; Mander et al., 2013, 2014; Nazir et al., 2013; Morgado et al., 2015; Needham et al., 2015; Mander and Punyasena, 2016). However, other studies using light microscopy (LM) have shown morphometric differences in the size of the Poaceae pollen grain species (Salgado-Labouriau and Rinaldi, 1990; Katsiotis and Forsberg, 1995; Joly et al., 2007; Schüler and Behling, 2011a,b; Jan et al., 2014). In addition, when analyzing large sets of palynomorphs from quaternary sediments, the use of SEM is a difficult and time-consuming task. Thus, morphometric datasets may be valuable for use in studies of fossil Poaceae pollen analysis (Schüler and Behling, 2011a,b; Jan et al., 2014).

**Table 1:** **Dataset of Poaceae analyzed species, type of microscopy used and vegetation type**.

**Vegetation type**	**Microscopy type**	**Number of species**	**Reference**
Grassland	SEM	5	Ahmad et al., 2011
Grassland	LM	2	Bauermann et al., 2013
Grassland	LM/SEM	4	Chaturvedi and Datta, 2001
Grassland	SEM	2	Chaturvedi et al., 1994
Grassland	LM/SEM	19	Chaturvedi et al., 1998
Grassland/Forest	LM/SEM	30	Côrrea et al., 2005
Grassland	LM/SEM	6	Datta and Chaturvedi, 2004
Grassland/Forest	SEM	86	Dórea, 2011
Grassland/Forest	LM	16	Heusser, 1971
Grassland	LM	160	Jan et al., 2014
Grassland	LM	35	Joly et al., 2007
Grassland	LM/SEM	2	Kashikar and Kalkar, 2010
Grassland	LM	11	Katsiotis and Forsberg, 1995
Grassland	SEM	12	Köhler and Lange, 1979
Grassland	LM/SEM	1	Linder and Ferguson, 1985
Grassland	SEM	57	Liu et al., 2004
Grassland	LM/SEM	1	Liu et al., 2005
Grassland/Forest	SEM	19	Mander and Punyasena, 2016
Grassland	SEM	12	Mander et al., 2013
Grassland	SEM	12	Mander et al., 2014
Grassland/Forest	LM	17	Markgraf and D'Antoni, 1978
Grassland	LM	9	Medeanic et al., 2008
Grassland	LM	3	Melhem et al., 2003
Grassland	LM/SEM	45	Morgado et al., 2015
Grassland/Forest	LM	6	Nakamura et al., 2010
Grassland	LM/SEM	4	Nazir et al., 2013
Grassland	LM/SEM	31	Needham et al., 2015
Grassland	LM/SEM	54	Perveen and Qaiser, 2012
Grassland	LM/SEM	20	Perveen, 2006
Grassland	LM	3	Radaeski et al., 2011
Grassland	LM	1	Radaeski et al., 2014a
Grassland	LM	6	Radaeski et al., 2014b
Grassland/Forest	LM	64	Roubik and Moreno, 1991
Grassland/Forest	LM	49	Salgado-Labouriau and Rinaldi, 1990
Grassland	LM	—(fossil pollen)	Schüler and Behling, 2011a
Grassland	LM	—(fossil pollen)	Schüler and Behling, 2011b
Grassland/Forest	SEM	11	Skvarla et al., 2003
Grassland	LM	1	Tedesco et al., 1999
Grassland	LM	3	Wilberger et al., 2004

Recently, studying pollen grains of fossil Poaceae in the grassland ecosystems of South America, Schüler and Behling (2011a) discovered potential new ways to distinguish grassland types. In their later study, Schüler and Behling (2011b) were able to differentiate the ecosystems present in South America. Moreover, Jan et al. (2014) succeeded in identifying a pattern among changes in the size of Poaceae pollen grains according to the ploidy level and C_3_ and C_4_ metabolism, thereby demonstrating that polyploid species have a larger pollen grain size. C_4_ species are tropical and inhabit warmer and drier regions, while temperate Poaceae species are C_3_ and live in humid and cold conditions (Boldrini, 2006). C_3_ and C_4_ species are important for paleoclimate studies because they indicate past variation in temperature and precipitation (Schüler and Behling, 2011a).

The aim of the study was to distinguish Poaceae pollen grains from grassland and forest vegetation of southern Brazil. The pollen grains of 68 species were analyzed to answer the following questions: (1) Can Poaceae pollen grains be separated into those of grassland species and those of forest species? (2) Do the pollen grains of forest species differ in size according to their arboreal or herbaceous habit?

## Materials and methods

### Collection of botanical material

During the field expeditions, 98 specimens of Poaceae were obtained, anticipating the 21 taxa representatives of this family, and some pollen material, being fertile, was selected for extraction. To obtain hibernal and estival plants in the flowering seasons, the samples were gathered using the transversal method (Filgueiras et al., 1994) in winter, autumn, spring, and summer in May, August, September, October, November, and December of 2013, as well as in January of 2014.

**Figure 2:**
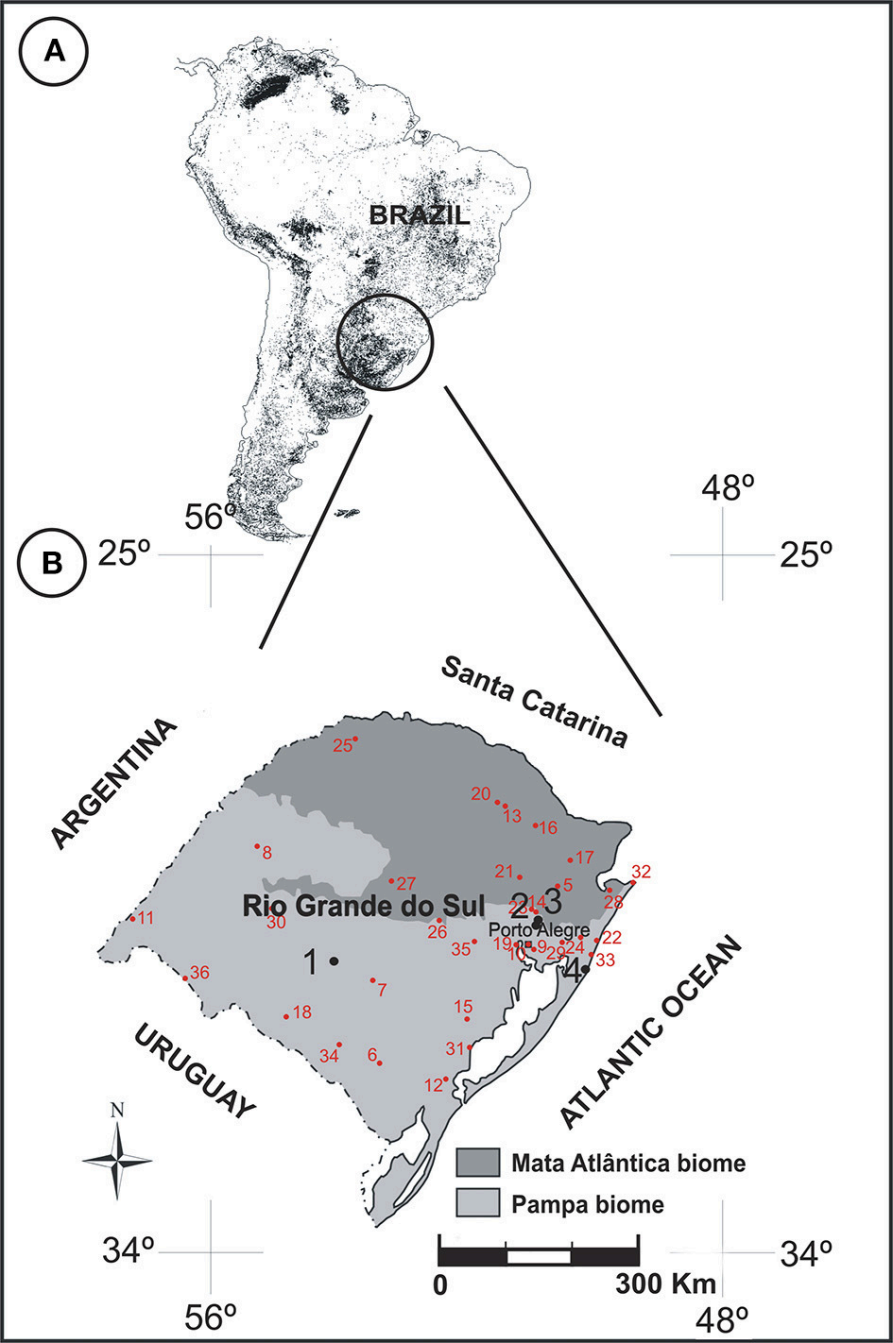
**(A)** Distribution of grasslands in South America highlighting the southern region of Brazil (adapted from Eva et al., 2002). **(B)** Map of Rio Grande do Sul showing the sampling sites in RS (black circles): 1. “Cerro do Ouro,” São Gabriel city; 2. Cachoeirinha city; 3. “Sitio Laranjal,” Gravataí city; 4. “Balneário Quintão,” Palmares do Sul city. Red circles indicate regions of collection of herbarium species (see Table 2 for more details of the names of regions).

After collection, the plants were pressed and dehydrated. The plants were identified by a skilled taxonomist (A. A. Schneider). The collection of herbarium specimens was deposited in the “Herbário do Museu de Ciências Naturais” from the Universidade Luterana do Brasil (MCNU/HERULBRA), and duplicates were deposited in the “Herbário Bruno Edgar Irgang” from Unipampa (HBEI/UNIPAMPA). The anthers were collected for chemical treatment of the herbarium materials from other Poaceae species. Since some species have state-restricted distribution, or sporadic bloom periods, samples were collected from pollen material in accordance with information provided by the ICN Herbarium (Figure 2, Table 2).

**Table 2:** **Information of the examined material in the Rio Grande do Sul, Brazil**.

**Species**	Collection site–Number^*^	**Collector**
*Agrostis* sp. L.	São Francisco de Paula–5	Z. Rúgolo, H. Longhi-Wagner, S. Boechat and A.M. Molina 1435
*Aira elegans* Willd. ex Gaudin	Pinheiro Machado–6	I. I. Boldrini 1143
*Amphibromus quadridentulus* (Döll) Swallen	São Francisco de Paula–5	Longhi-Wagner, Boldrini, and Miotto 2654
*Andropogon* cf. *lindmanii* Hack.	Palmares do Sul–4	J. N. Radaeski
*Andropogon lateralis* Nees	Caçapava do Sul–7	S. G. Bauermann
*Aristida* sp. L.	Itacurubi–8	S. G. Bauermann
*Arundinella hispida* (Humb. and Bonpl. ex Willd.) Kuntze	Viamão–9	R. Trevisan and Boldrini 830
*Axonopus* sp. P. Beauv	Itacurubi–8	S. G. Bauermann
*Bothriochloa laguroides* (DC.) Herter	Porto Alegre–10	M. Marchi 97
*Bouteloua megapotamica* (Spreng.) Kuntze	Uruguaiana–11	H. S. A. 74
*Bromus catharticus* Vahl	Cachoeirinha–2	J. N. Radaeski
*Calamagrostis viridiflavescens* (Poir.) Steud	Pelotas–12	I. Gabino 20
*Catapodium rigidum* (L.) C.E.Hubb	Porto Alegre–10	J. Valls
*Chascolytrum subaristatum*(Lam.) Desv	São Gabriel–1	J. N. Radaeski
*Chloris canterae* Arechav	Uruguaiana–11	J. Valls and A. Barcellos 2477
*Chusquea juergensii* Hack.	Passo Fundo–13	J. Valls, H. Longhi-Wagner, and A. Barcellos 3081
*Colanthelia cingulata* (McClure and L.B.Sm.) McClure	Araricá–14	R. Schmidt and Ene
*Cynodon dactylon* (L.) Pers	Gravataí–3	J. N. Radaeski
*Dactylis glomerata* L.	Porto Alegre–10	J. Valls
*Danthonia montana* Döll	Cristal–15	A. Guglieri, F. J. M. Caporal, S. Mochiutti, and M. Behling 533
*Digitaria ciliares* (Retz.) Koeler	Vacaria–16	B. Irgang and M. L. Porto
*Eleusine tristachya* (Lam.) Lam	Cachoeirinha–2	J. N. Radaeski
*Elionurus candidus* (Trin.) Hack.	Porto Alegre–10	R. Setubal 235
*Eragrostis bahiensis* Schrad. ex Schult	Gravataí–3	J. N. Radaeski
*Eragrostis neesii* Trin	São Gabriel–1	J. N. Radaeski
*Eustachys distichophylla* (Lag.) Nees	Porto Alegre–10	R. Setubal 683
*Festuca fimbriata* Nees	Jaquirana–17	I. Boldrini 1636
*Glyceria multiflora* Steud	Dom Pedrito–18	Valls, Gonçalves, Salles, and Moraes 6959
*Guadua trinii* (Nees) Nees ex Rupr	Guaíba–19	N. I. Matzembacker 2293
*Gymnopogon spicatus* (Spreng.) Kuntze	Lagoa Vermelha–20	Boldrini, Pillar, Kafpel, and Jacques 334
*Holcus lanatus* L.	Caxias do Sul–21	K. Hagelund 3797
*Hordeum stenostachys* Godr	Dom Pedrito–18	H. Longhi-Wagner 1560
*Ichnanthus pallens* (Sw.) Munro ex Benth	Guaíba–19	V. Citadini 59
*Imperata brasiliensis* Trin	Tramandaí–22	Waechter 1019
*Ischaemum minus* J.Presl	Gravataí–3	J. N. Radaeski
*Leersia* sp. Sol. ex Sw	Dois Irmãos–23	H. M. Longhi-Wagner
*Leptochloa fusca* (L.) Kunth	Osório–24	J. Valls et al. 4760
*Lithachne pauciflora* (Sw.) P.Beauv	Tenente Portela–25	Valls, Lindeman, Irgang, Oliveira, and Pott 1782
*Luziola peruviana* Juss. ex J.F.Gmel	Porto Alegre–10	Lacê
*Melica* sp. L.	São Gabriel–1	J. N. Radaeski
*Merostachys multiramea*Hack	Santa Cruz do Sul–26	V. Kinupp
*Microchloa indica*(L.f.) P. Beauv	Porto Alegre–10	H.M. Longhi-Wagner and C.A.D. Welker 9757a
*Muhlenbergia schreberi*J.F.Gmel	Estrela Velha–27	R. Trevisan
*Olyra latifolia*L.	Morrinhos do Sul–28	L. C. Mancino, T. B. Guimarães, L. R. M. Batista, and G. E. Ferreira
*Panicum aquaticum*Poir	Capivari do Sul–29	E. N. Garcia 892
*Pappophorum philippianum* Parodi	São Francisco de Assis–30	E. Freitas 359
*Parodiolyra micrantha* (Kunth) Davidse and Zuloaga	Gravataí–3	J. Valls, J. Jung, and A. M. Barcellos 2151
*Paspalum notatum* Flüggé	Caçapava do Sul–7	S. G. Bauermann
*Paspalum pauciciliatum* (Parodi) Herter	Gravataí–3	J. N. Radaeski
*Paspalum urvillei* Steud	Palmares do Sul–4	J. N. Radaeski
*Phalaris angusta* Nees ex Trin	São Lourenço do Sul–31	C. Bonilha 486
*Pharus lappulaceus* Aubl	Gravataí–3	L. R. M. Baptista
*Piptochaetium montevidense* (Spreng.) Parodi	São Gabriel–1	J. N. Radaeski
*Poa bonariensis* (Lam.) Kunth	Vacaria–16	A. Kappel
*Polypogon elongatus* Kunth	Torres–32	A. Barcelos and B. Irgang 9
*Schizachyrium microstachyum* (Desv. ex Ham.) Roseng	Caçapava do Sul–7	S. G. Bauermann
*Setaria parviflora* (Poir.) Kerguélen	Gravataí–3	J. N. Radaeski
*Spartina ciliata* Brongn	Cidreira–33	H. M. Longhi-Wagner and S. Leite
*Sporobolus indicus* (L.) R.Br	Gravataí–3	J. N. Radaeski
*Stipa filifolia* Nees	Bagé–34	H. M. Longhi-Wagner 5042
*Stipa melanosperma* J. Presl	São Francisco de Paula–5	R. L. C. Bortoluzzi 816
*Stipa papposa* Nees	Bagé–34	I. Boldrini 1177
*Stipa setigera* J.Presl	Bagé–34	S. C. Boechat
*Streptochaeta spicata* Schrad. ex Nees	Torres–32	J. F. M. Valls 1055
*Tridens brasiliensis* (Nees ex Steud.) Parodi	Cachoeira do Sul–35	J. Valls
*Trachypogon filifolius* (Hack.) Hitchc	Quaraí–36	Boldrini and Pilz187
*Tripogon spicatus* (Nees) Ekman	Quaraí–36	Boldrini, Barreto, Boechat and Pillar 279

* *Numbers corresponding to the Map of Figure 2B*.

The collection of pollen materials from a herbarium (containing plants from different regions of the state) enabled the authors of this study to establish an overview of the modern grass pollen of RS. By combining the findings of this study with the work of Hasenack et al. (2010) on the vegetation physiognomy of the state, we were able to establish a relationship between the pollen and the regional flora. Only 50 Poaceae species of four subfamilies comprise forest vegetation. Thus, we considered the selection of 10 forest species from all tribes and subfamilies for pollen analysis to be adequate. More species of grassland (58 species) were analyzed because there are a greater number of Poaceae species (450 species) in southern Brazil (Boldrini and Longhi-Wagner, 2011) than forest species.

### Treatment and description of pollen grains

The anthers were chemically processed according to the acetolysis methodology proposed by Erdtman (1952). After acetolysis, using glycerinated jelly, five permanent slides were created for each sample and deposited in the Laboratório de Palinologia da ULBRA. The pollen grains were measured and the slides mounted on the same day to prevent any changes in pollen size (Salgado-Labouriau, 2007). Schüler and Behling (2011a) measured 60 pollen grains from fossil pollen samples, but in this paper we studied modern pollen grains, measuring 25 pollen grains according to the methodology used for the study of modern pollen grains (Erdtman, 1952; Barth and Melhem, 1988).

**Figure 3:**
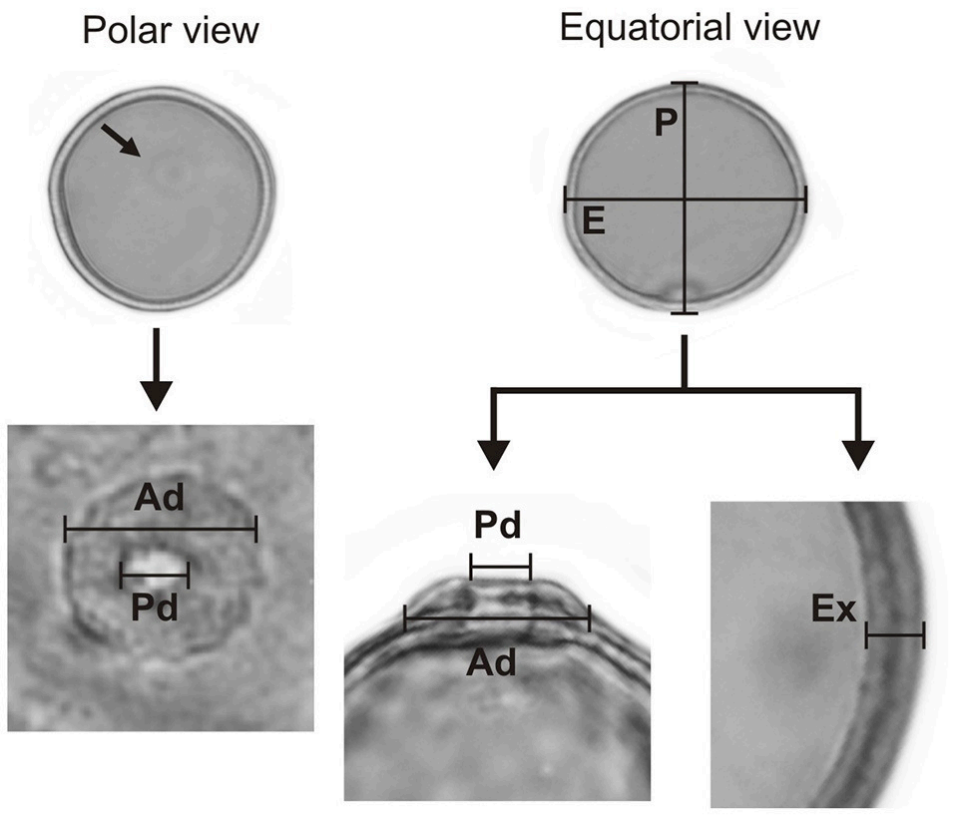
**The parameters of a Poaceae pollen grain are considered in the present paper**. Polar diameter (P), equatorial diameter (E), pore diameter (Pd), annulus diameter (Ad), and exine thickness (Ex).

The morphological characteristics of the pollen grains were observed and described by LM. A Leica CME microscope was used for measurements and for recording images. Using a × 1000 magnification, we recorded the polar diameter (P), equatorial diameter (E), or only the diameter (D) of spherical pollen grains, and the thickness of the exine (Ex) in 25 randomly selected pollen grains. In addition to the above, measurements of the pore and annulus width of the studied species were recorded (Figure 3). The pollen grains were then described in regard to their pollen unit, size, symmetry, polarity, amb, type of aperture, and ornamentation, using the terminology proposed by Barth and Melhem (1988) and Punt et al. (2007).

### Statistical analysis

BioEstat 5.0 and PAST 3.05 software was used for the statistical analysis. BioEstat 5.0 software was used to compile a frequency distribution histogram of pollen grain sizes. The histogram was then constructed from the size of the pollen grains of species inhabiting arboreous forest, grassland, and herbaceous forest. This program (BioEstat 5.0) was also used to determine size differences among pollen grains from arboreous forest, grassland, and herbaceous forest species using One-way ANOVA followed by Tukey's test. PAST 3.05software was used for discriminant analysis (DA) of separate groups according to the size of the pollen grains. In DA we use the minimum, maximum, and average size of pollen grains of all species to determine whether they can be grouped. We used this program (PAST 3.05) also to show the correlation between pollen grain, annulus, and pore sizes by Pearson correlation. The Pearson's correlation coefficient test was used to determine the average size of the pollen grains, the pore width, and the annulus width for all species (68 species studied). The PAST 3.05 software was also used to construct a box plot. The box plot shows overlapping measures and measures that do not overlap. We used all the pollen grain size measurements for the box plot (i.e., 25 measures each of 68 species = total 1700 measures).

## Results

### Measurement of pollen grains

**Figure 4:**
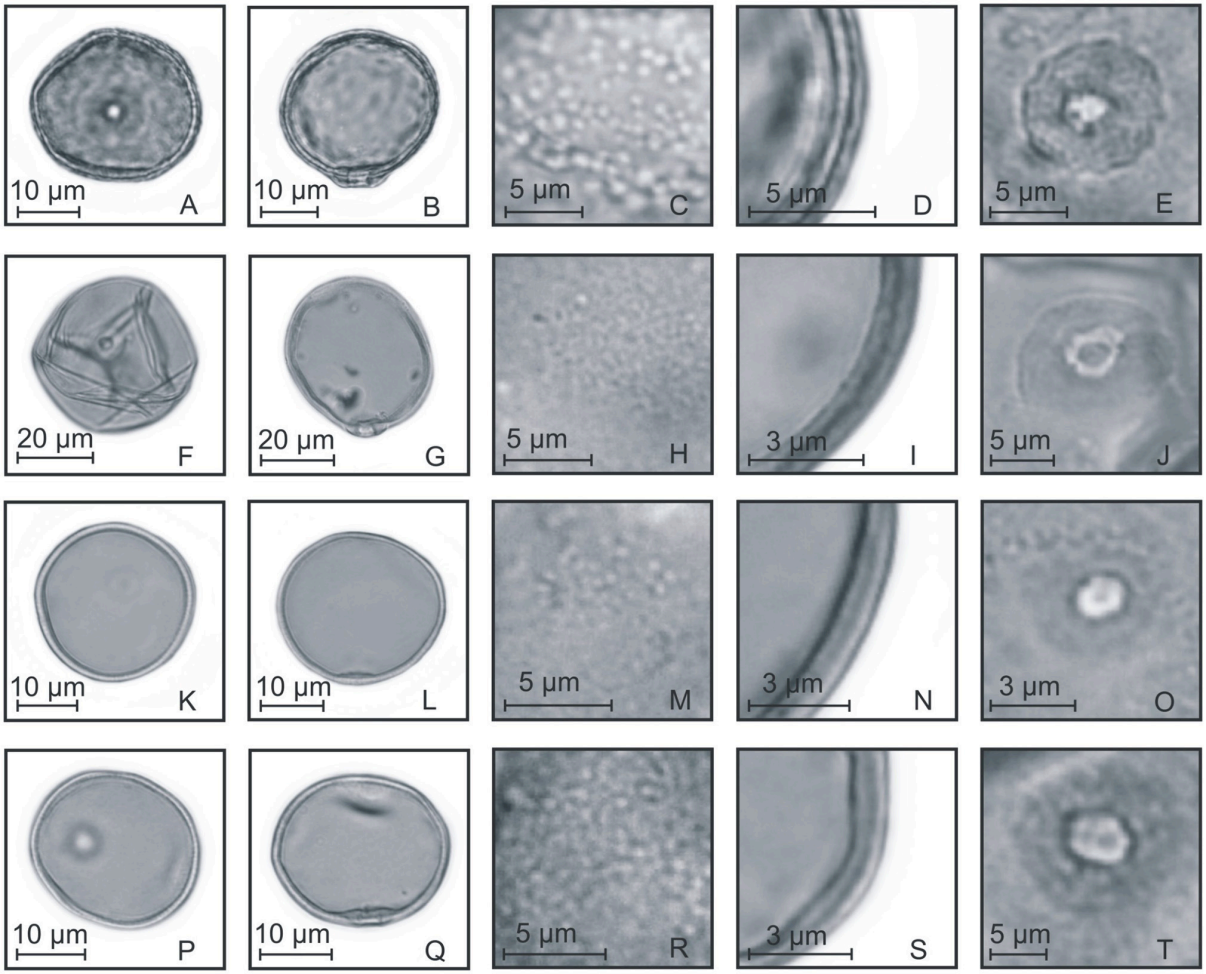
**Pollen grains of the subfamilies Anomochlooideae, Bambusoideae, and Pharoideae. (A–E)** *Streptochaeta spicata*: PV **(A)**, EV **(B)**, detail of ornamentation **(C)**, detail of the thickness of the exine **(D)**, and detail of the aperture **(E)**; **(F–J)** *Chusquea juergensii*: PV **(F)**, EV **(G)**, detail of ornamentation **(H)**, detail of the thickness of the exine **(I)**, and detail of the aperture **(J)**; **(K–O)** *Olyra latifolia*: PV **(K)**, EV **(L)**, detail of ornamentation **(M)**, detail of the thickness of the exine **(N)**, and detail of the aperture **(O)**; **(P–T)** *Pharus lappulaceus*: PV **(P)**, EV **(Q)**, detail of ornamentation **(R)**, detail of the thickness of the exine **(S)**, and detail of the aperture **(T)**.

**Figure 5:**
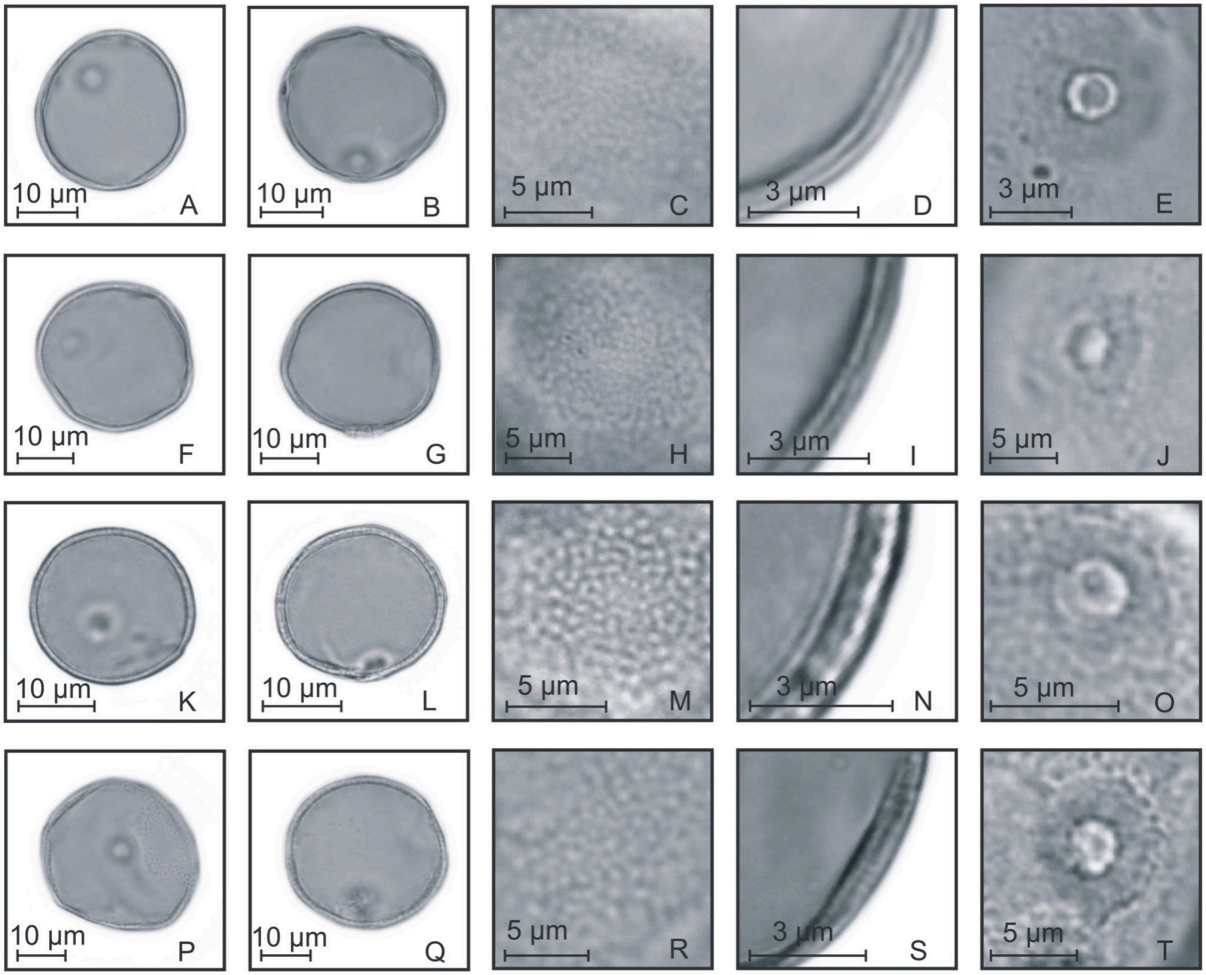
**Pollen grains of the subfamilies Ehrarthoideae, Danthonioideae, and Chloridoideae. (A–E)** *Leersia* sp.: PV **(A)**, EV **(B)**, detail of ornamentation **(C)**, detail of the thickness of the exine **(D)**, and detail of the aperture **(E)**; **(F–J)** *Danthonia montana*: PV **(F)**, EV **(G)**, detail of ornamentation **(H)**, detail of the thickness of the exine **(I)**, and detail of the aperture **(J)**; **(K–O)** *Eragrostis neesii*: PV **(K)**, EV **(L)**, detail of ornamentation **(M)**, detail of the thickness of the exine **(N)**, and detail of the aperture **(O)**; **(P–T)** *Chloris canterae*: PV **(P)**, EV **(Q)**, detail of ornamentation **(R)**, detail of the thickness of the exine **(S)**, and detail of the aperture **(T)**.

**Figure 6:**
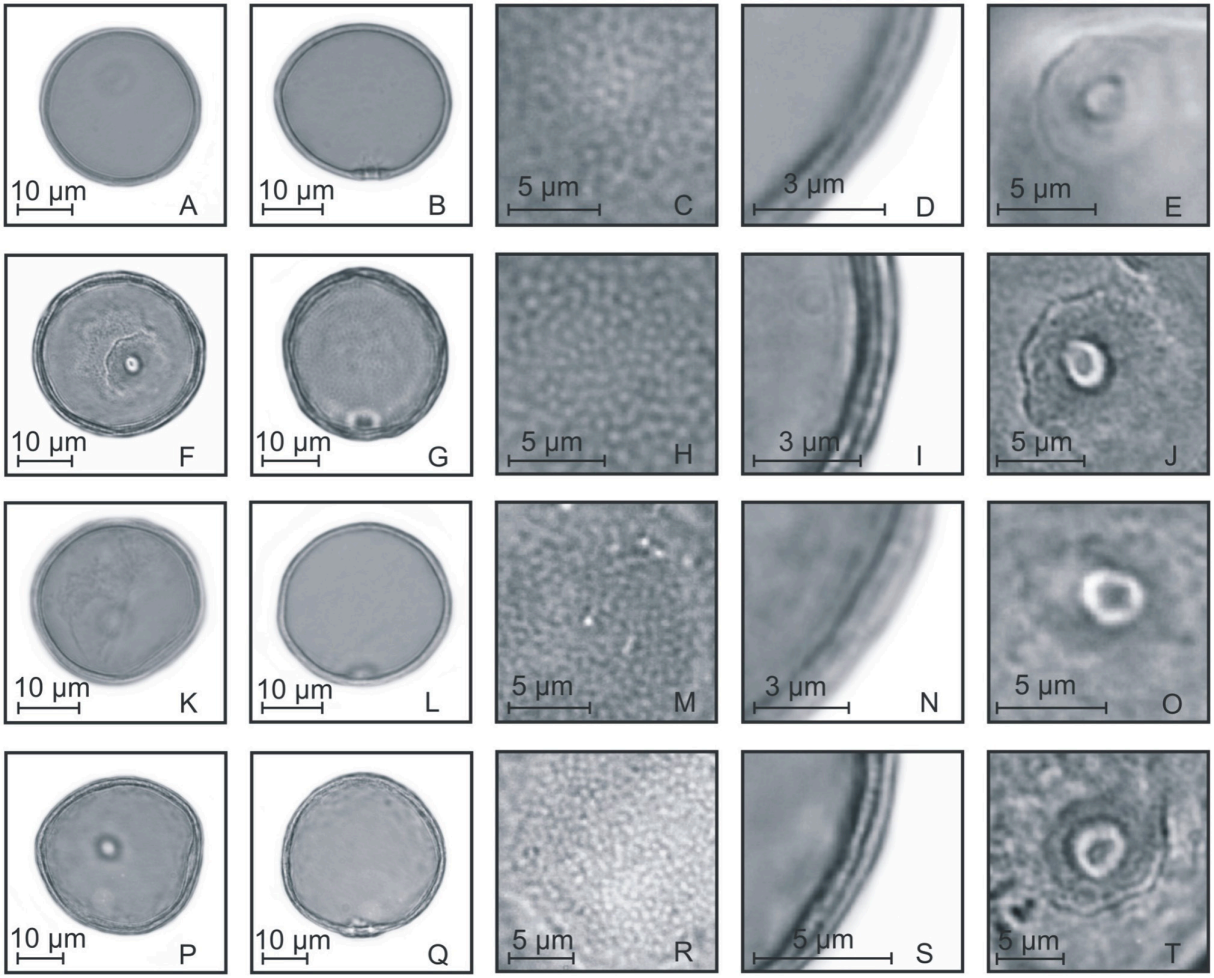
**Pollen grains of the subfamilies Chloridoideae, Aristidoideae, and Pooideae. (A–E)** *Pappophorum philippianum*: PV **(A)**, EV **(B)**, detail of ornamentation **(C)**, detail of the thickness of the exine **(D)**, and detail of the aperture **(E)**; **(F–J)** *Aristida* sp.: PV **(F)**, EV **(G)**, detail of ornamentation **(H)**, detail of the thickness of the exine **(I)**, and detail of the aperture **(J)**; **(K–O)** *Chascolytrum subaristatum*: PV **(K)**, EV **(L)**, detail of ornamentation **(M)**, detail of the thickness of the exine **(N)**, and detail of the aperture **(O)**; **(P–T)** *Bromus catharticus*: PV **(P)**, EV **(Q)**, detail of ornamentation **(R)**, detail of the thickness of the exine **(S)**, and detail of the aperture **(T)**.

**Figure 7:**
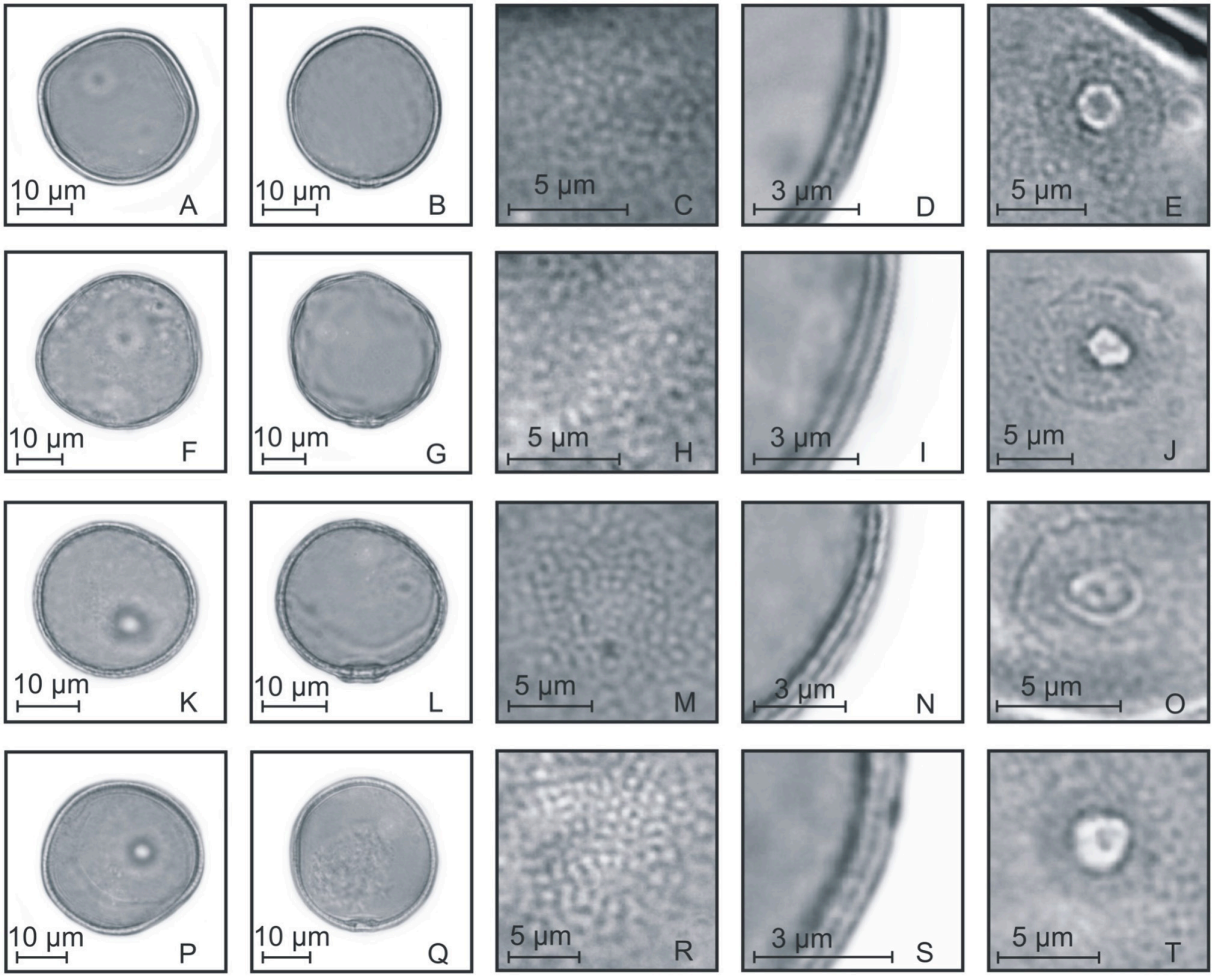
**Pollen grains of the subfamilies Pooideae and Panicoideae. (A–E)** *Melica* sp.: PV **(A)**, EV **(B)**, detail of ornamentation **(C)**, detail of the thickness of the exine **(D)**, and detail of the aperture **(E)**; **(F–J)** *Hordeum stenostachys*: PV **(F)**, EV **(G)**, detail of ornamentation **(H)**, detail of the thickness of the exine **(I)**, and detail of the aperture **(J)**; **(K–O)** *Piptochaetium montevidense*: PV **(K)**, EV **(L)**, detail of ornamentation **(M)**, detail of the thickness of the exine **(N)**, and detail of the aperture **(O)**; **(P–T)** *Paspalum urvillei*: PV **(P)**, EV **(Q)**, detail of ornamentation **(R)**, detail of the thickness of the exine **(S)**, and detail of the aperture **(T)**.

**Figure 8:**
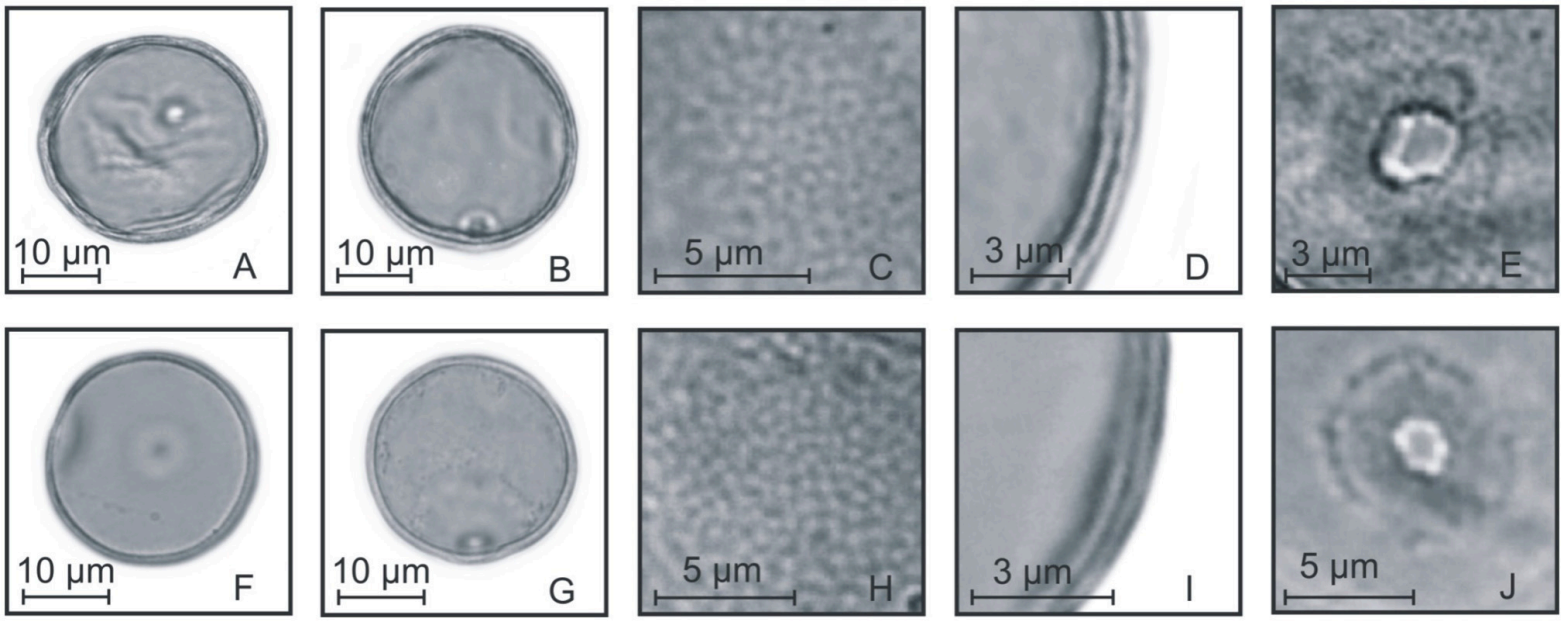
**Pollen grains of the subfamily Panicoideae. (A–E)** *Schizachyrium microstachyum*: PV **(A)**, EV **(B)**, detail of ornamentation **(C)**, detail of the thickness of the exine **(D)**, and detail of the aperture **(E)**; **(F–J)** *Arundinella hispida*: PV **(F)**, EV **(G)**, detail of ornamentation **(H)**, detail of the thickness of the exine **(I)**, and detail of the aperture **(J)**.

Table 3 presents the measurements of the pollen grains for the 68 species, in evolutionary order according to the GPWG (Grass Phylogeny Working Group) classification (2001). Yet, differences were observed in the measurements of pollen grains, pores, and annulus. Pollen grains of species of each tribe were selected to show the Poaceae morphological characteristics of all the tribes of southern Brazil (Figures 4–8).

**Table 3:** **Pollen morphological measurements of 68 Poaceae species in the region of southern Brazil**.

**Subfamily**	**Tribe**	**Species**	**Vegetation**	**Size**	**Diameter pollen grain (μm)**	**Aperture**	**Pore diameter (μm)**	**Annulus diameter (μm)**	**Exine (μm)**	**Figs**.
Anomochlooideae	Streptochaeteae	*Streptochaeta spicata*	Forest	Medium	28 (23–32)	monoporate	3 (2–4)	9 (8–10)	1.32	3A-E
Bambusoideae	Bambuseae	*Chusquea juergensii*	Forest	Large	44 (40–52)	monoporate	5 (4-6)	12 (11-13)	1.08	3F-J
		*Colanthelia cingulata*	Forest	Large	58 (47–74)	monoporate	5 (4–6)	14 (13–15)	1.32	
		*Guadua trinii*	Forest	Large	60 (50–77)	monoporate	5 (4–6)	14 (13–15)	1.52	
		*Merostachys multiramea*	Forest	Large	50 (44–55)	monoporate	5 (4–6)	14 (13–15)	1.52	
	Olyreae	*Lithachne pauciflora*	Forest	Medium	27 (25–28)	monoporate	2 (1–3)	7 (6–8)	1	
		*Olyra latifolia*	Forest	Medium	27 (23–30)	monoporate	2 (1–3)	6 (5–7)	1.08	3K-O
		*Parodiolyra micrantha*	Forest	Medium	30 (26–37)	monoporate	3 (2–4)	9 (8–10)	1	
Pharoideae	Phareae	*Pharus lappulaceus*	Forest	Medium	25 (23–27)	monoporate and diporate	3 (2–4)	8 (7–9)	1.08	3P-T
Ehrarthoideae	Oryzeae	*Leersia* sp.	Grassland	Medium	27 (23–34)	monoporate	2 (1–3)	7 (6–8)	1	4A-E
		*Luziola peruviana*	Grassland	Medium	26 (24–30)	monoporate	2 (1–3)	6 (5–7)	1.08	
Danthonioideae	Danthonieae	*Danthonia montana*	Grassland	Medium	28 (22–32)	monoporate	3 (2–4)	8 (7–9)	1	4F-J
Chloridoideae	Eragrostideae	*Eragrostis neesii*	Grassland	Small	22 (19–26)	monoporate	2,5 (2–3)	5 (4–6)	1	4K-O
		*Eleusine tristachya*	Grassland	Medium	28 (23–33)	monoporate	3 (2–4)	8 (7–9)	1	
		*Eragrostis bahiensis*	Grassland	Medium	29 (22–33)	monoporate	3 (2–4)	8 (7–9)	1	
		*Leptochloa fusca*	Grassland	Medium	25 (21–28)	monoporate	2 (1–3)	6 (5–7)	1	
		*Muhlenbergia schreberi*	Grassland	Medium	30 (26–35)	monoporate	2 (1–3)	6 (5–7)	1.04	
		*Sporobolus indicus*	Grassland	Small	22 (18–26)	monoporate	2 (1–3)	6 (5–7)	1	
		*Tridens brasiliensis*	Grassland	Medium	33 (30–36)	monoporate	3 (2–4)	8 (7–9)	1.1	
		*Tripogon spicatus*	Grassland	Medium	25 (20–27)	monoporate	2 (1–3)	6 (5–7)	1	
	Cynodonteae	*Bouteloua megapotamica*	Grassland	Medium	34 (25–38)	monoporate	3 (2–4)	9 (8–10)	1	
		*Chloris canterae*	Grassland	Medium	33 (27–37)	monoporate	3 (2–4)	8 (7–9)	1.04	4P-T
		*Cynodon dactylon*	Grassland	Medium	28 (24–32)	monoporate	3 (2–4)	8 (7–9)	1.04	
		*Eustachys distichophylla*	Grassland	Medium	30 (25–35)	monoporate	2 (1–3)	7 (6–8)	1.08	
		*Gymnopogon spicatus*	Grassland	Medium	34 (29–39)	monoporate	3 (2–4)	9 (8–10)	1.12	
		*Microchloa indica*	Grassland	Medium	25 (22–30)	monoporate	2 (1–3)	6 (5–7)	1	
		*Spartina ciliata*	Grassland	Medium	34 (32–37)	monoporate	3 (2–4)	8 (7–9)	1	
	Pappophoreae	*Pappophorum philippianum*	Grassland	Medium	30 (25–36)	monoporate	3 (2–4)	7 (6–8)	1	5A–E
Aristidoideae	Aristideae	*Aristida* sp.	Grassland	Medium	31 (26–33)	monoporate	3 (2–4)	8 (7–9)	1.08	5F-J; 8A,B
Pooideae	Poeae	*Agrostis* sp.	Grassland	Medium	29 (26–34)	monoporate	3 (2–4)	9 (8–10)	1	
		*Aira elegans*	Grassland	Small	22 (16–25)	monoporate	2 (1–3)	6 (5–7)	1.04	
		*Amphibromus quadridentulus*	Grassland	Medium	35 (32–38)	monoporate	3 (2–4)	9 (8–10)	1	
		*Calamagrostis viridiflavescens*	Grassland	Medium	28 (24–32)	monoporate	3 (2–4)	9 (8–10)	1.04	
		*Catapodium rigidum*	Grassland	Small	24 (22–27)	monoporate	2 (1–3)	6 (5–7)	1	
		*Chascolytrum subaristatum*	Grassland	Medium	29 (21–32)	monoporate	3 (2–4)	8 (7–9)	1.1	5K-O
		*Dactylis glomerata*	Grassland	Medium	33 (29–37)	monoporate	3 (2–4)	9 (8–10)	1.04	
		*Festuca fimbriata*	Grassland	Medium	35 (28–39)	monoporate	3 (2–4)	9 (8–10)	1	
		*Glyceria multiflora*	Grassland	Medium	36 (33–39)	monoporate	3 (2–4)	10 (9–11)	1	
		*Holcus lanatus*	Grassland	Medium	25 (23–28)	monoporate	2 (1–3)	6 (5–7)	1	
		*Poa bonariensis*	Grassland	Medium	28 (25–32)	monoporate	3 (2–4)	9 (8–10)	1	
		*Phalaris angusta*	Grassland	Medium	35 (32–39)	monoporate	3 (2–4)	9 (8–10)	1	
		*Polypogon elongatus*	Grassland	Medium	32 (27–37)	monoporate	3 (2–4)	8 (7–9)	1	
	Bromeae	*Bromus catharticus*	Grassland	Medium	37 (32–43)	monoporate	3 (2–4)	9 (8–10)	1.2	5P-T
	Meliceae	*Melica* sp.	Grassland	Medium	30 (25–33)	monoporate	2 (1–3)	7 (6–8)	1	6A-E
	Triticeae	*Hordeum stenostachys*	Grassland	Medium	37 (33–40)	monoporate	3 (2–4)	9 (8–10)	1	6F-J
	Stipeae	*Piptochaetium montevidense*	Grassland	Medium	27 (23–29)	monoporate	3 (2–4)	9 (8–10)	1.04	6K-O; 8C
		*Stipa filifolia*	Grassland	Medium	30 (27–35)	monoporate	3 (2–4)	9 (8–10)	1.04	
		*Stipa melanosperma*	Grassland	Medium	38 (34–39)	monoporate	3 (2–4)	10 (9–11)	1	
		*Stipa papposa*	Grassland	Medium	28 (24–35)	monoporate	3 (2–4)	9 (8–10)	1.04	
		*Stipa setigera*	Grassland	Medium	31 (25–34)	monoporate	3 (2–4)	9 (8–10)	1.04	
Panicoideae	Paniceae	*Axonopus* sp.	Grassland	Medium	29 (22–37)	monoporate	3 (2–4)	7 (6–8)	1	
		*Digitaria ciliares*	Grassland	Medium	37 (34–40)	monoporate and diporate	3 (2–4)	9 (8–10)	1.04	
		*Ichnanthus pallens*	Forest	Small	23 (22–26)	monoporate	2 (1–3)	6 (5–7)	1	
		*Panicum aquaticum*	Grassland	Medium	35 (30–40)	monoporate	3 (2–4)	9 (8–10)	1.24	
		*Paspalum notatum*	Grassland	Medium	34 (32–39)	monoporate	2,5 (2–3)	6 (5–7)	1.2	
		*Paspalum pauciciliatum*	Grassland	Medium	42 (37–46)	monoorate and diporate	3 (2-4)	8 (7–9)	1.04	
		*Paspalum urvillei*	Grassland	Medium	33 (29–36)	monoporate	3 (2–4)	9 (8–10)	1	6P-T
		*Setaria parviflora*	Grassland	Medium	34 (30–37)	monoporate	3 (2–4)	8 (7-9)	1.2	
		*Steinchisma hians*	Grassland	Medium	24 (18-27)	monoporate	2 (1–3)	6 (5–7)	1	
	Andropogoneae	*Andropogon lateralis*	Grassland	Medium	32 (28–36)	monoporate	4 (3–5)	9 (8–10)	1.2	
		*Andropogon* cf. *lindmanii*	Grassland	Medium	38 (34–41)	monoporate	3 (2–4)	9 (8–10)	1	
		*Bothriochloa laguroides*	Grassland	Medium	36 (33–38)	monoporate	3 (2–4)	8 (7–9)	1.2	
		*Elionurus candidus*	Grassland	Medium	35 (28–39)	monoporate	3 (2–4)	9 (8–10)	1.04	
		*Imperata brasiliensis*	Grassland	Medium	36 (33–39)	monoporate	3 (2–4)	9 (8–10)	1.04	
		*Ischaemum minus*	Grassland	Medium	33 (30–37)	monoporate	3 (2–4)	8 (7–9)	1.04	
		*Schizachyrium microstachyum*	Grassland	Medium	30 (24–36)	monoporate	3 (2–4)	9 (8–10)	1.3	7A-E
		*Trachypogon filifolius*	Grassland	Medium	37 (31–42)	monoporate	3 (2–4)	9 (8–10)	1.24	
	Arundinelleae	*Arundinella hispida*	Grassland	Medium	25 (21–30)	monoporate	2 (1–3)	7 (6–8)	1	7F-J

**Figure 9:**
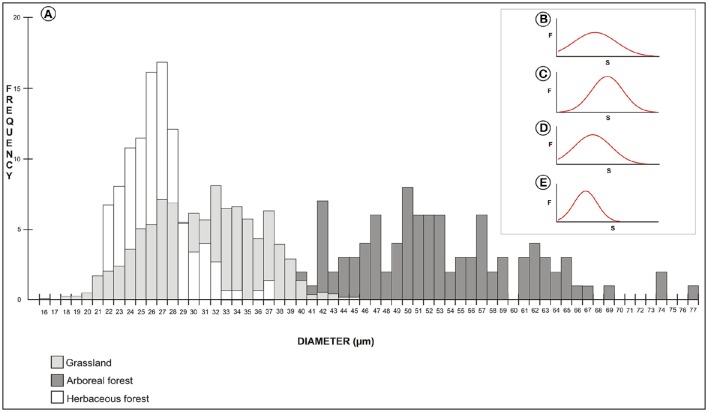
**Frequency distribution histogram of the pollen size measurements (A)**. Gaussian distribution of size measurements of pollen grains of arboreous species **(B)**, grassland species **(C)**, herbaceous forest species **(D)**, and all species studied **(E)**. F, frequency; S, pollen grain size.

A frequency distribution histogram of the measurements of pollen size is shown in Figure 9A. The average measurement values were higher for arboreous forest (8% of the measurements of these species were 50 μm). Grassland and herbaceous forest species had lower average measurement values (16% of the forest herbaceous species measured 27 μm, and 8% of the grassland species measured 32 μm). Grain size distributions showed a Gaussian distribution for samples of arboreous forest, grassland, and herbaceous forest species (Figures 9B–E). The Gaussian distribution showed that ANOVA-Tukey can be applied to the data set.

### Morphometric variation in diameters of the pollen grains, pores, and annulus

The ANOVA-Tukey test showed statistically significant differences between the size of pollen grains of arboreous forest, grassland, and herbaceous forest species (Table 4). This difference is clear in the comparison of samples that indicate values (*p*) less than 0.01. The difference between the means of the arboreous forest and grassland samples was large (22.1719), while among the grassland and herbaceous forest samples the difference was small (4.3681).

**Table 4:** **Significances between the size of pollen grains of forest arboreous, grassland, and forest herbaceous species obtained with ANOVA-Tukey**.

**Sources of variation**	**DF**	**Sum of squares**	**Mean squares (variances)**
Treatments	2	50.6 e + 03	25.3 e + 03
error	1672	45.0 e + 03	26.924
F =	939.0160		
(*p*) =	<0.0001		
Mean (arboreal forest pollen grains)	53.0800		
Mean (grassland pollen grains)	30.9081		
Mean (herbaceous forest pollen grains)	26.5400		
Tukey:	Diference	S	(p)
Means (arboreal forest–grassland)	22.1719	58.4142	<0.01
Means (arboreal forest–herbaceous forest)	26.5400	56.0297	<0.01
Means (grassland–herbaceous forest)	4.3681	13.8690	<0.01

**Figure 10:**
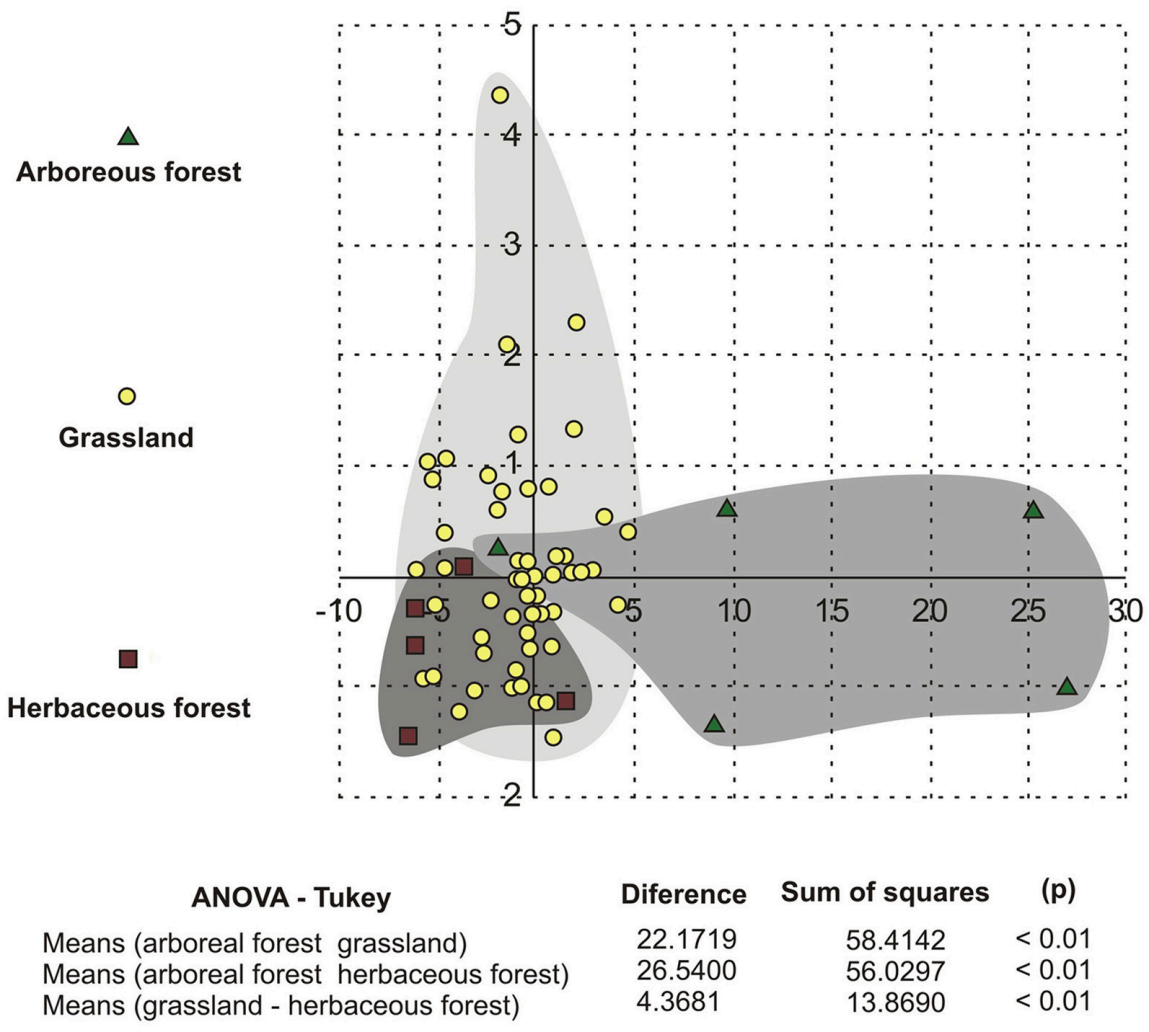
**Discriminant analysis of the pollen grain size ordination of the 68 Poaceae species**.

The DA test (Figure 10) determined the separation of three groups according to the values of the variables. The DA identified the separation of arboreous forest grassland and herbaceous forest groups. The arboreous forest group showed the greatest differences, and the grassland and herbaceous forest groups were mixed.

**Figure 11:**
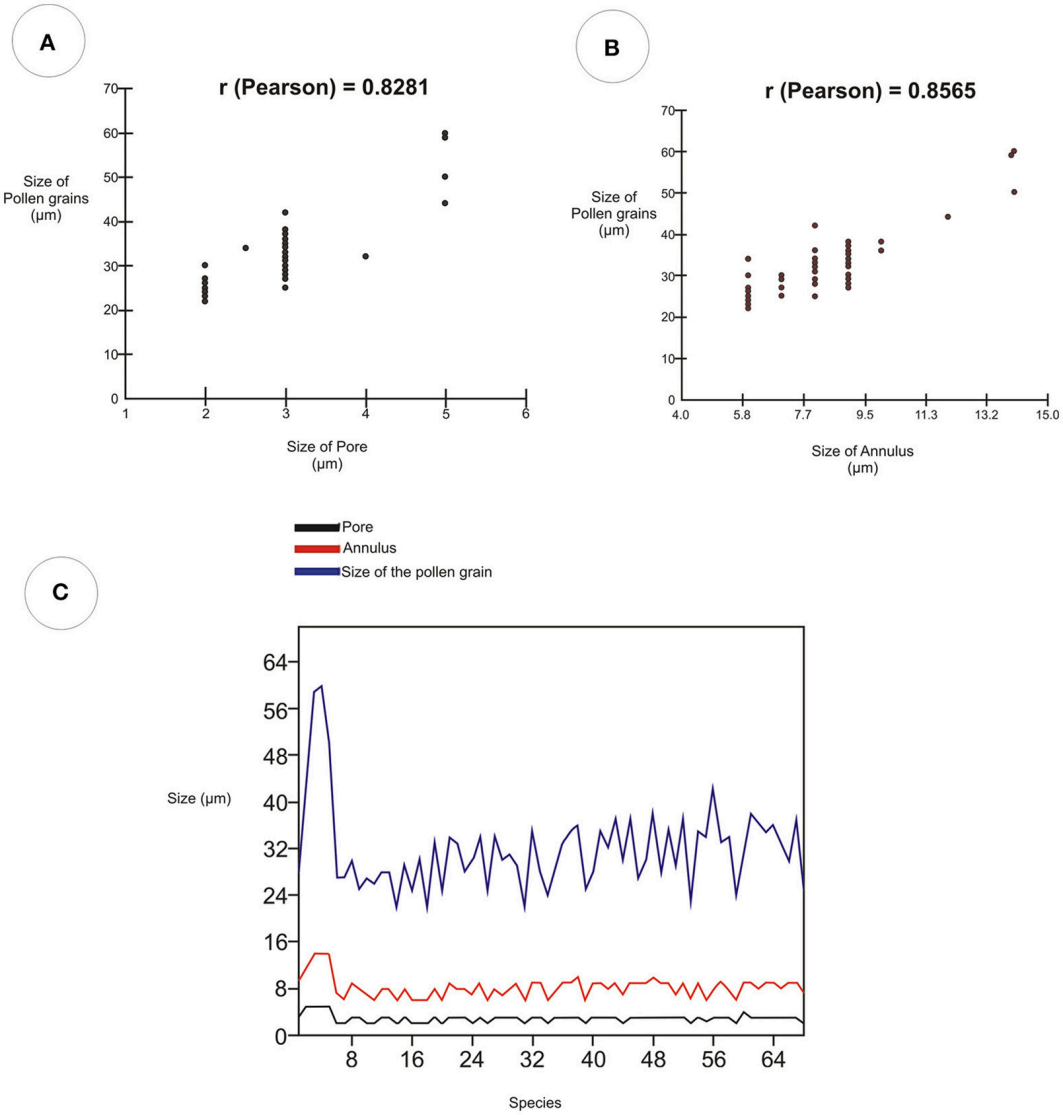
**Pearson correlation diagram showing the strength of the relationship between pore, annulus, and size of pollen grain for the 68 Poaceae species. (A)** Correlation between the variable sizes of the pore and size of the pollen grain. **(B)** Correlation between the variable annulus sizes and the pollen grain sizes. **(C)** Linear chart of the values of the diameters of the pores, annulus, and pollen grains.

The Pearson correlation test (Table 5) showed values that indicate a strong relationship between the size of pollen grains and the width of the pore (*r* = 0.8281). It also showed a strong relationship between the size of pollen grains and the width of the annulus (*r* = 0.8565). The diagrams of the values obtained indicate a similarity between the size of pollen grains, the pore width, and the width of the annulus (Figure 11).

**Table 5:** **Pearson correlation coefficient values showing the strength of relationship among the pore, annulus and size of the pollen grain**.

	**Pore–annulus**	**Pore–pollen grain**	**Annulus–pollen grain**
*n* =	68	68	68
r (Pearson) =	0.9257	0.8281	0.8565
IC 95% =	0.88–0.95	0.73–0.89	0.78–0.91
IC 99% =	0.86–0.96	0.70–0.91	0.74–0.92
R2 =	0.8569	0.6858	0.7336
*t* =	19.8779	12.0019	13.4797
(*p*) =	<0.0001	<0.0001	<0.0001

All taxa showed monoporate apertures with annulus around the pores, except for *Pharus lappulaceus, Digitaria ciliares*, and *Paspalum pauciciliatum*, which showed diporate as well as the monoporate pollen grains. However, these three species (*P. lappulaceus, D.ciliares*, and *P. pauciciliatum*) showed only a few diporate pollen grains; most of their pollen grains were found to be monoporate. Nevertheless, they were unique species in terms of having diporate pollen. In the herbaceous forest species with diporate pollen grains, the grains measured 23–27 μm in width, while in the grassland species with diporate pollen, the grains measured 34–46 μm in width.

### Interpretation of, and distinction between, the grassland and forest pollen grains

In southern Brazil, 80% of Poaceae species are grassland species, while 20% are forest species. In our data set (68 species), 85.29% were grassland species and 14.71% were forest species. Thus, we analyzed the appropriate proportions of species relating to grassland and forest vegetation in the region.

**Figure 12:**
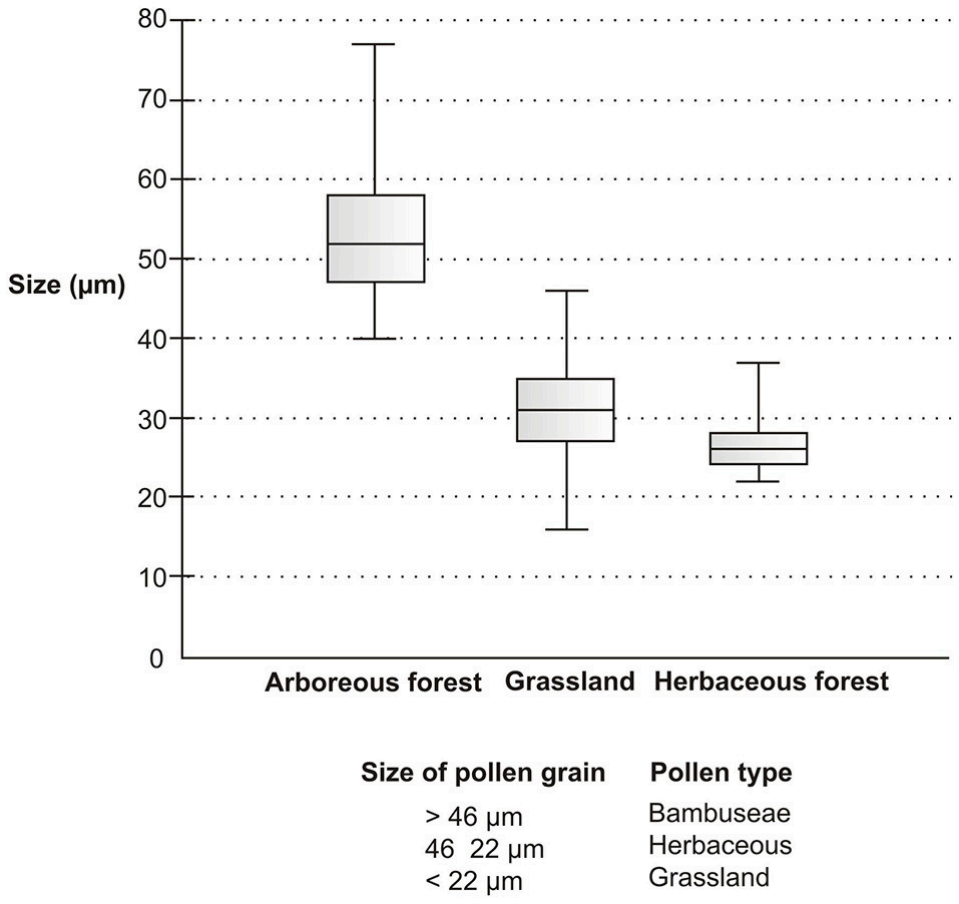
**Chart box plot of the diameters of the pollen grains**. The bold horizontal line within the box represents the median. The box shows 50% of the interquartile range, and whiskers the total variation.

The box plot of data sets relating to different Poaceae vegetation (arboreous forest, grassland, and herbaceous forest) showed pollen grains of different size ranges (Figure 12). The pollen grains of arboreous forest species were larger than those of grassland and herbaceous forest species. The pollen grains of grassland species and herbaceous forest species were found to be of similar size. However, the pollen of the grassland species had a lower minimum size than that of the forest herbaceous species. Three pollen types could be separated based on pollen grain size (Table 6). The Bambuseae pollen type was found to have pollen grains larger than 46 μmin width. Pollen grains that vary in size between 22 and 46 μm are of the herbaceous pollen type; these pollen grains belong to either grassland or herbaceous forest species. The grassland pollen type has small pollen grains, measuring less than 22 μm.

**Table 6:** **Pollen measures and establishment of pollen types**.

**Size of pollen grain**	**Pollen type**	**Species included**
>46 μm	Bambuseae	*Chusquea juergensii, Colanthelia cingulata, Guadua trinii, Merostachys multiramea*
46–22 μm	Herbaceous	*Streptochaeta spicata, Lithachne pauciflora, Olyra latifolia, Parodiolyra micrantha, Pharus lappulaceus, Leersia* sp., *Luziola peruviana, Danthonia montana, Eleusine tristachya, Eragrostis bahiensis, Muhlenbergia schreberi, Tridens brasiliensis, Bouteloua megapotamica, Chloris canterae, Cynodon dactylon, Eustachys distichophylla, Gymnopogon spicatus, Microchloa indica, Spartina ciliata, Pappophorum philippianum, Aristida* sp., *Agrostis* sp., *Amphibromus quadridentulus, Calamagrostis viridiflavescens, Catapodium rigidum, Dactylis glomerata, Festuca fimbriata, Glyceria multiflora, Holcus lanatus, Poa bonariensis, Phalaris angusta, Polypogon elongatus, Bromus catharticus, Melica* sp., *Hordeum stenostachys, Piptochaetium montevidense, Stipa filifolia, Stipa melanosperma, Stipa papposa, Stipa setigera, Axonopus* sp., *Digitaria ciliares, Ichnanthus pallens, Panicum aquaticum, Paspalum notatum, Paspalum pauciciliatum, Paspalum urvillei, Setaria parviflora, Andropogon lateralis, Andropogon* cf. *lindmanii, Bothriochloa laguroides, Elionurus candidus, Imperata brasiliensis, Ischaemum minus, Schizachyrium microstachyum, Trachypogon filifolius*
<22 μm	Grassland	*Eragrostis neesii, Leptochloa fusca, Sporobolus indicus, Tripogon spicatus, Aira elegans, Chascolytrum subaristatum, Steinchisma hians, Arundinella hispida*

## Discussion

Based on measurements of pollen grains, previous studies have allowed scholars to distinguish between Poaceae pollen grains of South American ecosystems, and also to show the trends in pollen grain size among C_3_ and C_4_ Poaceae species (Schüler and Behling, 2011a,b; Jan et al., 2014). In this work, it was possible to distinguish the Poaceae pollen grains relating to grassland and forest species of southern Brazil. Jan et al. (2014) analyzed a large data set with species from various locations around the world. In our work we wanted to analyze the variability within one ecosystem; therefore, we chose to analyze a large set of data relating to only one region (southern Brazil).

Studies of Poaceae pollen grains have revealed a strong correlation between size of pollen grain, pore, and annulus (Skvarla et al., 2003; Joly et al., 2007; Schüler and Behling, 2011a,b; Jan et al., 2014). The results of our own study also showed a relationship between size of pollen, pore, and annulus, as determined through correlation analysis.

**Figure 13:**
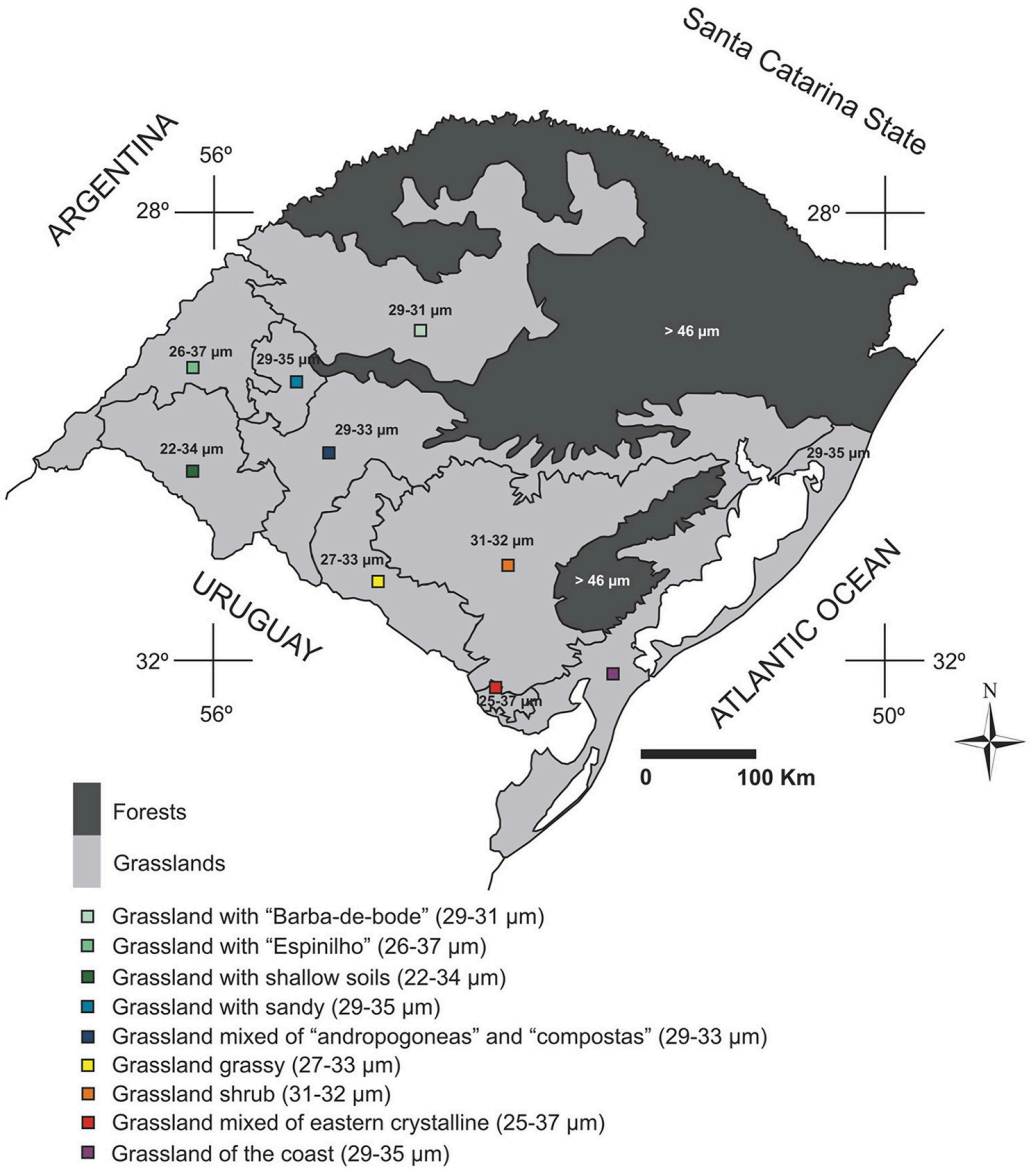
**Main changes in the average size of the Poaceae pollen grains in the different regions of Rio Grande do Sul, according to the vegetation physiognomies in RS (adapted from Hasenack et al., 2010)**.

Analysis of the Poaceae pollen of the plants deposited in the herbarium provided a description of the variation in pollen grain size of species that occur in different regions of the state of RS. According to the results, relating pollen data to information on the current vegetation of RS, the main variations in size of Poaceae pollen grains in the state (Figure 13) could be mapped. Taking into account the vegetation types that are based on more representative genera from different regions (Hasenack et al., 2010), we can assign to regions the probable main pollen types occurring in different locations. Thus, the northern half of RS seems to be composed of larger pollen grains. We also found a reduction in size toward the southern half of the state, where the concentration of smaller pollen grains can be associated with the western part of RS, especially in the region of grassland with shallow soils (where the range of diameters for pollen grains is 22–34 μm).

### Forest vegetation

Pollen grains of forest Poaceae species showed distinctions between species. Arboreous species showed larger pollen grains than herbaceous species. The pollen grain size of the arboreous species ranged from medium to large, while that of the herbaceous species ranged from small to medium. The pollen grains of arboreous Poaceae species showed a tendency toward larger sizes (Markgraf and D'Antoni, 1978; Salgado-Labouriau and Rinaldi, 1990). The differently sized grains of pollen forest species may be related to the small wind flow inside the forests and may also be influenced by pollination (Dórea, 2011). Some variations in the size of the pollen grains of modern Poaceae species has already been reported in South America. In Venezuela, larger pollen grains have already been reported to be related to the Bambusoideae and Pooideae subfamilies (Salgado-Labouriau and Rinaldi, 1990). However, in southern Brazil, we are able to differentiate at the level of tribes determining the Bambuseae pollen type. The Bambuseae pollen type is indicative of arboreal grasses and humid regions (Schmidt and and Longhi-Wagner, 2009).

### Grassland vegetation

Pollen grains of grassland species are smaller than those of arboreous forest species and similar to those of herbaceous forest species. These results make it possible to identify the herbaceous pollen type. The smaller size of pollen grains in grassland species allows identification of the grassland pollen type. The small and medium sizes of pollen grains of grassland Poaceae species correspond to previous data relating to South American species (Heusser, 1971; Markgraf and D'Antoni, 1978; Salgado-Labouriau and Rinaldi, 1990; Melhem et al., 2003; Côrrea et al., 2005; Bauermann et al., 2013; Radaeski et al., 2014a,b) and species from other regions of the world (Joly et al., 2007; Jan et al., 2014; Morgado et al., 2015). The small size of pollen grains of grassland species can also be related to the type of dispersion involved, since grassland species produce more pollen than forest species (Radaeski and Bauermann, 2016).

### Exine

Many studies have revealed differences in the exine sculpture of Poaceae pollen grains. Such differences are evident through the use of SEM, which allows adequate analysis of the surface (Köhler and Lange, 1979; Linder and Ferguson, 1985; Chaturvedi et al., 1994, 1998; Chaturvedi and Datta, 2001; Skvarla et al., 2003; Datta and Chaturvedi, 2004; Liu et al., 2004, 2005; Perveen, 2006; Kashikar and Kalkar, 2010; Ahmad et al., 2011; Dórea, 2011; Perveen and Qaiser, 2012; Mander et al., 2013, 2014; Nazir et al., 2013; Morgado et al., 2015; Needham et al., 2015; Mander and Punyasena, 2016). Light microscopy is used to study the fossil pollen of Quaternary sediments at smaller magnifications (× 400); SEM is not suitable for such study. Thus, data pollen measures seem to be more suitable to use in comparison with fossil pollen.

The thin exine of the Poaceae pollen grains is a remarkable characteristic, not exceeding 2 μm in thickness and having equivalent sexine and nexine values. Because of this thin layer, many pollen grains—especially the larger ones—may display small changes in their spherical shape owing to the flattening of the pollen grain. This often provides the impression of pollen grains with prolate or oblate forms. However, these shapes are easily observed in crushed pollen grains, for when the non-deformed (used as parameters) pollen grains are examined, their spherical form—characteristic of the Poaceae family—is noted.

The surface of the exine of Poaceae pollen grains, when viewed by SEM, exhibits several variations among species (Dórea, 2011). However, when observed under light microscopy, the pollen surface exhibits a tectate exine with columellae and spinulose ornamentation. The variations in the surface of the exine observed by SEM cannot be observed by light microscopy. To identify the pollen grains (from pollen records) involving smaller increases, mainly occurring in Quaternary sediments, the ornamentation is often not observed. With a magnification of × 400, much ornamentation of the studied taxa is not visible (Schüler and Behling, 2011a,b), for the ornamentation is often interpreted as psilate, scabrate, or microrreticulate surfaces. However, under higher (SEM) magnifications, the sculptured grain surfaces may be evident (Chaturvedi et al., 1998; Liu et al., 2004; Dórea, 2011; Mander et al., 2013).

## Conclusions

Using a data set of 68 species, we found that types of vegetation can be distinguished according to Poaceae pollen grains. The size of pollen grains of arboreous forest Poaceae species differs from that of grassland and herbaceous forest species. The pollen grains of forest species of arboreal habit are larger than those of forest species of herbaceous habit. Through measurements and statistical analysis, we found that these Poaceae species exhibit variation in the size of pollen grains in species inhabiting arboreous forest, grassland, and herbaceous forest. Thus, three pollen types were identified: Bambuseae, herbaceous, and grassland pollen types.

Grassland and forest vegetation may be distinguished by examining Poaceae pollen grains from southern Brazil. Thus, the dynamics of the grassland and forest vegetation during the Pleistocene and Holocene periods can be demonstrated based on Poaceae pollen grains. Also, pollen characterization by vegetation is of great importance, since the pollen morphology of the grasslands and forests can be used as indicators of humid or dry environments, respectively.

By determining the size of pollen grains of 68 Poaceae species in RS, it was possible to indicate previously inaccessible information for ecological inferences concerning southern Brazil grasslands. Attaining better taxonomic resolution of both vegetation types allows new opportunities to expand the pollen records beyond the family level. Further research is needed on the pollen morphology of other native genera and species of the Poaceae family for RS. It is expected that further studies will allow greater differentiation between groups and improved knowledge of pollen morphology at a family level. The presented method is being applied to the development of pollen records for southern Brazil and may favor climactic reconstruction of past environments and an evaluation of the dynamics of Quaternary grassland Poaceae vegetation.

## Author contributions

JR provided the images of the pollen grains and pollen measurements. JR, SB, AP structured and edited the manuscript during all phases. SB and AP supervised the project. JR and SB supported the paleoecological interpretations. JR and AP developed the botanical implications.

### Conflict of interest statement

The authors declare that the research was conducted in the absence of any commercial or financial relationships that could be construed as a potential conflict of interest.
